# Effect of Carotid Artery Stenting on Cognitive Function in Patients with Internal Carotid Artery Stenosis and Cerebral Lacunar Infarction: A 3-Year Follow-Up Study in China

**DOI:** 10.1371/journal.pone.0129917

**Published:** 2015-06-11

**Authors:** Zhang Yong Xia, Qin Jian Sun, Hua Yang, Ming Xia Zhang, Ru Ban, Ge Lin Xu, Ya Ping Wu, Le Xin Wang, Yi Feng Du

**Affiliations:** 1 Department of Neurology, Shandong Provincial Hospital Affiliated to Shandong University, Jinan, Shandong, 250021, PR China; 2 Department of Neurology, Liaocheng People's Hospital and Liaocheng Clinical School of Taishan Medical University, Liaocheng, Shandong, 252000, PR China; 3 Department of Neurology, the Third People’s Hospital of Liaocheng, Liaocheng, Shandong, 252000, PR China; 4 Department of Neurology, Jinling Hospital, Nanjing University School of Medicine, Nanjing, Jiangsu, 210002, PR China; 5 School of Biomedical Sciences, Charles Sturt University, Wagga Wagga, NSW 2678, Australia; St Michael's Hospital, University of Toronto, CANADA

## Abstract

**Background and Objectives:**

Carotid artery stenting (CAS) is an important therapeutic strategy for patients with carotid artery stenosis. However, the potential influence of CAS on cognitive function in patients with carotid artery stenosis and cerebral lacunar infarction has not been determined. This study investigated changes in cognitive function associated with CAS and the factors related to these changes.

**Methods:**

This prospective cohort study comprised 579 Chinese patients with cerebral lacunar infarction and carotid artery stenosis for whom CAS was indicated, and a matched control group of 552 healthy individuals. Cognitive function before CAS and at scheduled intervals from 6 months to 3 years was assessed with instruments that included the Mini-Mental State Examination (MMSE) and Montreal Cognitive Assessment (MoCA) scale. Potential factors that might affect cognitive function were analyzed via logistic regression.

**Results:**

The MMSE and MoCA scores of the patients before CAS were significantly lower than that of the control subjects. These scores were significantly higher 6 months after CAS and sustained or increased throughout the 3-year follow-up. Also significantly improved after CAS from baseline were scores for an alternating trail test, cube copying, clock-drawing, attention, and delayed recall in an auditory-verbal learning test. Logistic regression analyses showed that age greater than 65 y, little education, diabetes, and hypertension were independent risk factors for deteriorated MoCA scores 3 years after CAS.

**Conclusion:**

CAS was associated with significantly improved cognitive function in cerebral lacunar infarction patients with severe stenosis.

## Introduction

The morbidity and mortality of stroke remains high in both developed [1] and developing countries [2], despite advances in diagnostic and therapeutic strategies. Cerebral lacunar infarction (CLI), a small deep infarction of the brain due mainly to an occlusion of a penetrating artery, accounts for about a quarter of all ischemic strokes [3]. Initially, it was thought that CLI was the result of benign vascular lesions, and that patients generally had favorable clinical outcomes. However, we now know that the rate of future stroke events in CLI patients is similar to that of stroke patients [4, 5]. Moreover, recent studies have suggested that patients with CLI are at increased risk of cognitive disorders and dementia [6]. Strategies for early prevention of deterioration of cognitive function in CLI patients are urgently needed.

Carotid artery stenosis caused by atherosclerosis is an independent risk factor for ischemic cerebrovascular disease [7, 8], and has also been associated with impairment of cognitive function [9]. It is logical to suppose that treatment strategies targeting stenosis of the carotid artery may be effective for delaying the progression of cognitive dysfunction in patients with ischemic cerebrovascular disease.

As a minimally invasive procedure, carotid artery stenting (CAS) is an important therapeutic strategy in carotid artery stenosis [10, 11]. Recent studies have investigated the potential influence of CAS on cognitive function in patients with carotid artery stenosis [12–15] but some results are inconsistent [16, 17]. However, the sample sizes of these studies were generally small, and further studies are needed to provide convincing conclusions. To the best of our knowledge, no previous study has investigated the potential influence of CAS on cognitive function in CLI patients.

The present multicenter prospective cohort study investigated changes in cognition in patients suffering both CLI and severe internal carotid artery (ICA) stenosis after CAS and the factors related to these changes. The cognitive function of Chinese CLI patients was monitored after CAS by utilizing the Mini-Mental State Examination (MMSE) and Montreal Cognitive Assessment (MoCA) scale, and factors that may affect changes of cognitive function in these patients were analyzed.

## Methods

### Patients and study protocol

The study was designed as a prospective cohort study to evaluate the influence of CAS on cognition function in CLI patients. The ethics committees of Shandong Provincial Hospital Affiliated to Shandong University approved the study protocol before performance of the study. All of the included patients provided written consent.

### Patients

Recruited were patients suffering both CLI and severe ICA stenosis, with CAS indicated. The patients were enrolled at sub-centers of the China Interventional Stroke Registry [18] from March 2006 to February 2011. These facilities included Liaocheng People’s Hospital in Shandong Province, Third People’s Hospital of Liaocheng, Shandong Provincial Hospital, and Nanjing Military General Hospital.

Only patients who conformed to the following criteria were included: first onset of stroke within 1 week before enrollment; CLI confirmed by cerebral computed tomography (CT) or magnetic resonance (MR) imaging; unilateral or bilateral severe stenosis of the ICA, detected through digital subtraction angiography; and symptoms of cerebral ischemia related to ICA stenosis. Cerebral lacunar infarction was diagnosed in accordance with the National Fourth Revised Diagnostic Criteria for Cerebrovascular Disease of China in 1995 [19]. Carotid artery stenosis was defined based on the standard set by the North America Symptomatic Carotid Endarterectomy Collaborative Research Group, with digital subtraction angiography [20]. CAS was indicated for symptomatic arterial diameter stenosis of either-sided ICA ≥ 50%, or asymptomatic stenosis ≥ 70%.

Excluded from the study were patients with cognitive disorders caused by nonvascular factors, such as congenital mental retardation; inability to complete the scale check; comorbidities such as severe heart, liver, or kidney diseases or complicated with malignancy; mental disorders including anxiety and depression; severe blood diseases or severe hemorrhagic tendency and not able to receive antithrombotic therapy; contraindications to antiplatelet medications or statins; vasculopathy or fibromuscular dysplasia caused by acute aortic dissection inflammation or acute arteritis, moyamoya disease, or radiotherapy; intracranial hemorrhage, hemorrhagic stroke, intracranial tumors, or diagnosed as intracranial arteriovenous malformation within 1 month before the enrollment; long-term substance abuse; symptomatic coronary artery disease requiring percutaneous coronary intervention; surgery within 1 month before enrollment, or planned to receive surgery within the upcoming 3 months; contraindicated to heparin or anesthetics.

The control group consisted of healthy individuals recruited from the neurologic departments of the above centers, who visited for health screenings during the study period. Individuals in the control group were without any neurologic diseases, psychological disorders, or physical dysfunction as confirmed by CT or MR examination. The control group served as a baseline reference for cognitive function, to which patients in the test group were compared.

### Data collection

The collected demographic and clinical data of patients included age, gender, fasting blood glucose, triglyceride, total cholesterol, systolic blood pressure, diastolic blood pressure, diabetes mellitus, coronary artery disease, smoking and drinking habits, education level, and handedness. In addition, patients with potential ICA stenosis and CLI who were scheduled to receive CAS underwent the following cerebral and vascular imaging examinations: color ultrasonography of the carotid artery, cerebral CT angiography, cerebral MR, and cerebral arterial angiography. The purposes of the imaging examinations was to assess brain structure, exclude other intracranial diseases, and obtain information regarding the location, morphology, and composition of plaques and the formation of collateral arteries compensatory to the artery stenosis. According to the general physical condition of the patient, some received CT perfusion imaging or MR perfusion weighted imaging, to assess regional cerebral blood flow.

The analyzed potential risk factors for deteriorated MoCA scores 3 years after CAS were: age greater than 65 years, comorbidities of hypertension, DM, dyslipidemia, current smoking, alcohol drinking, and level of education. Little education was defined as ≤12 years of education. The diagnostic criteria for DM were 2 consecutive fasting plasma glucose ≥7.0 mmol/L or an oral glucose tolerance test 2-hour plasma glucose ≥11.1 mmol/L. Hypertension was defined as systolic blood pressure ≥140 mmHg and (or) diastolic blood pressure ≥ 90 mmHg.

### Imaging examination

All patients underwent carotid artery color Doppler, brain CTA, brain MRI and cerebral angiography to characterize the vascular stenosis’ position, length, plaque, and collateral compensation and to determine the structure of brain tissue and exclude other intracranial disorders. Some patients underwent CT perfusion imaging, MR perfusion weighted imaging, or positron emission tomography to evaluate the regional cerebral blood flow and brain metabolism, depending on the condition of the individual.

### Carotid artery stenting (CAS)

All of the patients received CAS treatment within 14 days of the onset of CLI. The CAS procedure was performed as previously described [21]. Briefly, after local anesthesia, an 8F catheter sheath was implanted into the right femoral artery via a Seldinger puncture [22], and angiography was performed to detect the stenosis of the artery. An 8F guiding catheter was placed into the common carotid artery after systemic heparinization. A protective umbrella was implanted and released, and a balloon was placed at the stenosed portion of the artery and pre-expanded. An Acculink carotid stent (Abbott, US) of suitable size was implanted and released. Finally, the protective devices were removed and another angiography was performed to confirm that the residual stenosis of the artery was < 30%.

### Neuropsychological tests

The cognitive function of the patients was assessed according to scores on the MMSE and MoCA at enrollment in the study, and at 1, 6, 12, 24, and 36 months after CAS. The baseline MMSE and MoCA scores in healthy controls were also determined. Applications of the MMSE and MoCA were performed in accordance with the guidelines of the Chinese Interventional Stroke Registry System, as previously described [18]. MMSE included 30 cognitive items such as orientation, memory, calculation, understanding, retelling, and naming. The highest possible MMSE score was 30. Patients with MMSE scores >27 were considered cognitively normal and patients with MMSE scores <27 were considered cognitively impaired.

The Chinese version of the MoCA was adopted for cognitive appraisal [23]. MoCA included 10 cognitive items: an alternating trail test, cube copying, clock-drawing, naming, attention, sentence repeating, verbal fluency, abstraction, auditory-verbal learning test (AVLT)-delayed recall, and orientation. The maximum score attainable on the MoCA is 30 points. Patients with scores >26 were considered cognitively normal and patients with MMSE scores < 26 were considered cognitively impaired. If the MoCA score of a patient after CAS was lower than before CAS, this situation was defined as a deteriorated MoCA score. The above cognitive tests were all performed by Neurology Deputy Chief physicians who had received systematic training. The minimum time interval between the MMSE and MoCA scoring was 1 hour.

### Statistical analyses

Continuous variables are presented as mean ± standard deviation, and categorical variables as numbers and frequencies. The independent *t*-test was applied for the comparisons of continuous variables, and the chi-squared or Fisher’s exact tests for categorical variables. Multiple logistic regression tests were performed to detect potential independent risk factors for deterioration of MoCA scores 3 years after CAS, including age > 65 y, hypertension, DM, dyslipidemia, current smoking, alcohol drinking, and educational levels.

All of the statistical analyses were performed using SPSS16.0 statistical software. A *P*-value < 0.05 was considered statistically significant.

## Results

### General characteristics of the included patients and healthy controls

Initially recruited into the study were 628 patients who fulfilled the inclusion criteria. Among them, 4 patients died after CAS (2 for sudden cardiac death at 7 and 19 months, respectively; 1 for cerebral hemorrhage at 21 months; and 1 due to car accident at 25 months), and 45 patients were lost during follow-up or failed to finish the cognitive assessment.

Finally, 579 CLI patients with unilateral or bilateral severe stenosis of the ICA were included in the analyses (Table 1). The participants in the control group (n = 552) had no neurologic or psychological disorders. Participants of the CLI and control groups were matched for potential risk factors of atherosclerosis, including gender, age, educational levels, blood lipids, prevalence of hypertension, diabetes mellitus (DM), and smoking habits (*P* all > 0.05).

**Table 1:** Baseline characteristics of CLI patients and controls.*

	LCI patients	Controls	χ^2^	*t*	*P*
Subjects, n	579	552	—	—	—
Male, n (%)	336 (58.0)	296 (53.6)	2.227	—	0.735
Age, y	64.33 ± 9.17	64.10 ± 8.92	—	0.427	0.669
Fasting blood glucose, mmol/L	7.11 ± 2.09	6.97 ± 1.96	—	1.162	0.245
Triglyceride, mmol/L	1.98 ± 0.72	2.03 ± 0.69	—	1.192	0.233
Total cholesterol, mmol/L	6.03 ± 1.58	5.92 ± 1.61	—	1.159	0.246
SBP, mmHg	149.33 ± 20.65	150.67 ± 19.48	—	1.123	0.261
DBP, mmHg	85.78 ± 12.19	87.02 ± 14.23	—	1.570	0.116
DM, n (%)	81 (14.0)	67 (12.1)	0.852	—	0.355
Hypertensive, n (%)	108 (18.7)	121 (21.9)	1.868	—	0.171
Coronary artery disease, n (%)	94 (16.2)	81 (14.7)	0.526	—	0.468
Current smoking, n (%)	135 (23.3)	120 (21.7)	0.402	—	0.525
Alcohol drinking, n (%)	121 (20.9)	148 (26.8)	0.013	—	0.908
Education, y	10.94 ± 2.91	11.03 ± 3.17	—	0.497	0.618
Left handed, n (%)	67 (11.6)	59 (10.7)	0.222	—	0.637

* All values are mean ± standard deviation, unless indicated otherwise.

### Effects of CAS on MMSE and MoCA scores in patients with ICA stenosis and CLI

For CLI patients scheduled for CAS, at baseline the mean scores of the MMSE, MoCA, alternating trail test, cube copying, clock drawing, and attention and AVLT-delayed recall were all significantly lower than the scores of the control group (*P* < 0.05, all; Table 2). Six months after CAS, the MMSE and MoCA scores of the CLI group were significantly improved. Three years after CAS, the MMSE and MoCA scores of the CLI group had improved further. Scores for the alternating trail test, cube-copying, clock-drawing, attention, and AVLT-delayed recall improved during follow-up after CAS (*P* < 0.05, all).

**Table 2:** MMSE and MoCA scores of CLI and healthy controls.

			LCI patients					
		Controls	Before CAS	1 month	6 months	1 year	2 years	3 years
MMSE		28.67 ± 1.72	27.79 ± 1.94 ^a^ *	27.98 ± 2.15 ^a^ *	28.38 ± 2.12 ^a^ * ^,^ ^b^ **	28.55 ± 1.98 ^b^ **	28.53 ± 2.03 ^b^ **	28.61 ± 1.89 ^b^ **
MoCA		20.91 ± 2.08	19.97 ± 2.17 ^a^ *	19.91 ± 1.99 ^a^ *	20.70 ± 2.31 ^b^ **	20.82 ± 2.18 ^b^ **	20.93 ± 2.41 ^b^ **	20.89 ± 2.03 ^b^ **
	Alternating trail test	0.67 ± 0.43	0.59 ± 0.50 ^a^ *	0.60 ± 0.59 ^a^ *	0.63 ± 0.42	0.63 ± 0.61	0.65 ± 0.44 ^b^ *	0.66 ± 0.48 ^b^ *
	Cube copying	0.66 ± 0.38	0.57 ± 0.67 ^a^ *	0.59 ± 0.87	0.60 ± 0.94	0.67 ± 0.85 ^b^ *	0.65 ± 0.41 ^b^ *	0.66 ± 0.38 ^b^ **
	Clock-drawing	1.81 ± 0.57	1.64 ± 0.38 ^a^ *	1.69 ± 0.45 ^a^ *	1.70 ± 0.79 ^a^ *	1.74 ± 0.74 ^b^ **	1.75 ± 0.96 ^b^ *	1.78 ± 0.83 ^b^ **
	Naming	2.46 ± 0.61	2.39 ± 0.84	2.39 ± 0.71	2.41 ± 0.47	2.40 ± 0.53	2.41 ± 0.50	2.41 ± 0.63
	Attention	4.31 ± 1.19	4.02 ± 1.48 ^a^ *	4.15 ± 1.33 ^a^ *	4.18 ± 1.16 ^b^ *	4.28 ± 1.62 ^b^ **	4.30 ± 1.54 ^b^ **	4.34 ± 1.49 ^b^ **
	Sentence repeating	1.41 ± 0.56	1.34 ± 0.64	1.35 ± 0.65	1.34 ± 0.68	1.38 ± 0.54	1.37 ± 0.69	1.38 ± 0.66
	Verbal fluency	0.35 ± 0.48	0.31 ± 0.39	0.32 ± 0.45	0.33 ± 0.51	0.34 ± 0.40	0.33 ± 0.38	0.34 ± 0.43
	Abstraction	0.68 ± 0.54	0.61 ± 0.72	0.61 ± 0.71	0.62 ± 0.67	0.63 ± 0.48	0.63 ± 0.62	0.64 ± 0.69
	AVLT-delayed recall	3.34 ± 1.16	3.09 ± 1.22 ^a^ *	3.16 ± 1.43 ^a^ *	3.25 ± 1.29 ^b^ *	3.29 ± 1.37 ^b^ **	3.32 ± 1.51 ^b^ **	3.32 ± 1.47 ^b^ **
	Orientation	5.71 ± 0.59	5.64 ± 0.74	5.65 ± 0.65	5.64 ± 0.62	5.65 ± 0.48	5.67 ± 0.55	5.70 ± 0.41

MMSE, Mini-Mental State Examination; MoCA, Montreal Cognitive Assessment; AVLT, Auditory–Verbal Learning Test; CLI, cerebral lacunar infarction; CAS, carotid artery stenting.

^a^, Compared with the controls.

^b^, Compared with the LCI patients before CAS.

*P < 0.05 and ** *P* < 0.01.

### Risk factors for deteriorated MoCA Scores 3 years after CAS in CLI patients

Of the analyzed potential risk factors for deteriorated MoCA scores 3 years after CAS, the multiple logistic regression analyses indicated that the potential independent risk factors were age > 65 y; little education; and hypertension (Table 3).

**Table 3:** Independent risk factors of deteriorated MoCA Scores 3 years after CAS in CLI patients: results of multi-variable adjusted logistic regression analysis.

Variables	B	SE	Wald	*P*	OR	95% CI
Age >65 years	1.617	0.637	8.381	0.009	5.038	1.687–15.045
Little education	1.057	0.474	4.752	0.033	2.877	1.113–7.436
DM	0.912	0.298	4.363	0.046	2.489	1.058–5.855
Hypertension	0.438	0.332	7.997	0.021	1.549	1.144–2.097

Degrees of freedom = 1, for all factors. CI, confidence interval; OR, odds ratio; SE, standard error.

## Discussion

The primary objective of this multicenter prospective cohort study was to determine the effect of CAS on cognition in Chinese CLI patients with ICA stenosis. Cognitive function in this study was evaluated using two validated scoring systems, the MMSE and MoCA. The MoCA scale has been widely applied in clinics as a standard for evaluation of cognitive function; it is accepted that the sensitivity of MoCA is higher than that of the MMSE scale [24, 25]. Thus, the MoCA may be more suitable for the cognitive evaluation of participants with early or mild cognitive dysfunction. The Chinese version of the MoCA was adopted in this research as more appropriate to the study population [26]. Cognitive function was measured using the MMSE and MoCA, before and intermittently after CAS for 36 months. Scores were evaluated relative to those of healthy individuals matched for age, gender, and atherosclerotic risk factors. The results indicated that the cognition of patients with severe stenosis of the ICA and CLI at baseline (before CAS) was significantly impaired compared with the controls.

Our finding that the baseline MMSE and MoCA scores for patients with severe ICA stenosis and CLI were significantly lower than those of the matched controls is not consistent with the results of previous studies [6, 27]. The earlier reports were that carotid stenosis had little association with dementia, but lacunar infarction had a significant association. This may be because either carotid artery stenosis or CLI, or both, may be related to an impairment of cognitive function, probably due to ischemia of brain tissue [27]. More importantly, in the present study, 6 months after the CAS procedure the cognitive function of these patients had significantly improved, and the benefit remained stable or improved during the 3 years of follow-up. These results are in accord with those of previous studies that were performed in populations without confirmed CLI but who underwent CAS [14, 15].

The potential mechanisms underlying the beneficial effects of CAS toward cognitive function in CLI patients with severe stenosis of the ICA may include the following. Firstly, CAS may improve cognitive function by improving or restoring cerebral perfusion. Indeed, it has been confirmed in both experimental [28] and clinical observations [29] that low perfusion of brain tissue and subsequent injury to the functional area is a major cause of cognitive dysfunction. Therefore, early restoration of the cerebral blood supply may attenuate or even reverse ischemic damage to the brain.

Secondly, the optimized CAS procedure has been associated with reduced risk of recurrent stroke by preventing the progression of local atherosclerotic lesions [30]. This suggests that the early performance of CAS in suitable patients may reduce the incidence of stroke-associated cognitive dysfunction. Given the benefits of CAS shown by the present study for patients with severe ICA stenosis and CLI, the exact mechanisms responsible for these benefits deserve further study.

Our secondary objective in the present study was to determine the factors that may affect changes in cognitive function after CAS in these patients. It has been reported previously that gender, older age, and little education are risk factors for cognitive deterioration, whereas whether hyperlipidemia, diabetes mellitus, smoking, or drinking are risk factors is controversial [31]. Most previous studies have been limited by small samples and short follow-up periods, and the conclusions are inconsistent not only for these reasons but by differences in the enrolled population, cognition scale, and education level. In the present study, we found that age over 65 years, hypertension or DM as comorbidities, and little education were independent predictors for deterioration of the MoCA score 3 years after the procedure. These results suggest that CAS performed after newly diagnosed CLI may delay the deterioration of cognitive function in patients with severe ICA stenosis. To the best of our knowledge, ours is the first study to observe the midterm effects of CAS in CLI patients.

There have been reports that the CAS procedure itself may be associated with deterioration of cognition [13], or that the influence of CAS on cognition is not always favorable [17]. From our perspective, the adverse effects of CAS on cognitive function may be more directly related to post-procedural complications such as injury due to ischemia-reperfusion or microembolization in the distal arteries [32]. Therefore, physicians should strive to lower the rate of complications associated with CAS through the proper selection of indicated patients, and high-quality perioperative care and medications. Most important for achieving the benefits of CAS on cognition is the selection of the optimal treatment strategy and experience of the physicians.

The results of our logistic analyses indicated that older age, comorbidities such as hypertension and diabetes, and little education may be responsible for the deterioration of the cognitive function in these patients. These results indicated that risk factors that were related to the progression of atherosclerosis may also accelerate the deterioration of cognitive function in CLI patients with ICA stenosis. Therefore, optimal pharmacologic treatment for the control of blood pressure and blood glucose may also be important for the delay of cognitive dysfunction in these patients.

The limitations of the study should be considered when interpreting the results. First, this was an observational cohort study, and the results should be confirmed in a randomized controlled trial. Secondly, although the MMSE and MoCA have been validated in previous studies to assess cognitive function, they may not reflect the overall cognitive function of patients. In addition, in this study regional blood was not evaluated to confirm an effective improvement in cerebral perfusion. Finally, the potential influence of the distribution and location of the infarct on the effect of CAS on cognitive function was not evaluated, because of the limited sample size. Future studies are needed to determine the patient subgroups who may benefit most from the CAS procedure with regard to cognitive function.

In conclusion, our results indicated that, 6 months after the procedure, CAS was associated with significantly improved cognitive function in CLI patients with severe stenosis of the ICA, and these benefits lasted for at least 3 years after the procedure. These results show that randomized controlled trials to evaluate the potential influence of CAS on cognitive function are warranted.
